# Fractal features of soil particle size distributions and their potential as an indicator of *Robinia pseudoacacia* invasion^1^

**DOI:** 10.1038/s41598-018-25543-0

**Published:** 2018-05-04

**Authors:** Kun Li, Huanxiang Yang, Xu Han, Lingyu Xue, Yang Lv, Jinhua Li, Zhanyong Fu, Chuanrong Li, Weixing Shen, Huiling Guo, Yikun Zhang

**Affiliations:** 1 Taishan Forest Ecosystem Research Station/Shandong Provincial Key Laboratory of Soil Erosion and Ecological Restoration, Taian, Shandong 271018 China; 2 Taishan Scenery and Scenic Spot Area Management Committee, Taian, Shandong 271000 China

## Abstract

To study the fractal dimensions of the soil particle size distributions (PSDs) within different plantations (of *Pinus densiflora, Quercus acutissima, Robinia pseudoacacia*, and *Larix kaempferi*) and evaluate PSDs as an indicator of the likelihood of *Robinia pseudoacacia* invasion, the soil porosity of 0–20 cm soil layers was measured at different plantations in the Yaoxiang Forest Farm, Shandong Province, China. The results showed that the fractal dimension (*D*_m_) values varied from 2.59 to 2.70 among the different plantations and were significantly negatively correlated to sand content and positively correlated to silt content and clay content. Significant negative correlations were observed between *D*_m_ and both soil organic matter (SOM) (*P* < 0.05) and available phosphorus (*P* < 0.01). The multifractal entropy dimension (*D*_1_) and entropy dimension/capacity dimension (*D*_1_/*D*_0_) parameters were not significantly correlated with SOM, although significant correlations were found between SOM and each of *D*_0_, Δ*α*, and Δ*f*(*α*). Compared with the other plantations, the *Robinia pseudoacacia* plantation had higher nutrient contents, higher *D*_0_ and *D*_1_ values and lower *D*_m_ values. Based on principal component analysis (PCA) ordination, we concluded that *Robinia pseudoacacia* and *Pinus densiflora* shared a similar habitat and that *Robinia pseudoacacia* is more likely to invade *Pinus densiflora* plantations for soil.

## Introduction

Soil is a porous medium with different particle compositions that display irregular shapes and self-similar structures, and it exhibits fractal characteristics. Therefore, fractal theory has been applied to the study of soil characteristics^1^. Texture is determined by the distribution of different particle size fractions and represents a fundamental characteristic of soil that has a profound influence on physical, chemical and biological processes^2^. The distribution equation of the particle weight/size distribution has been calculated as the fractal dimension of the soil particle size distribution, which can be used to characterize the size and uniformity of soil^3^ and can serve as a useful parameter for monitoring soil degradation induced by land-use patterns and changes^4^. Furthermore, the fractal dimension is a measure of the fragility of the fragmented material^5^. The mass fractal dimension of soil particles is one of the inherent properties of soil, and certain assumptions, such as uniform density, which have been questioned when calculating the soil-mass fractal dimension^6^. Therefore, the concept of the volume fractal dimension was developed, and a formula to calculate the soil volume fractal dimension was introduced^7^. The volume fractal dimension of soil particles is a fundamental characteristic of soil. Presently, research on soil particle size distributions (PSDs) is primarily focused on a single dimension^8,9^, and this topic has been well studied^10–12^. However, many studies with detailed experimental data have shown that a single fractal dimension is not sufficient to describe PSDs in soil^13^. To obtain more detailed information on soil PSDs, multifractal techniques have been adopted from information science into soil science^14^.

Multifractal and fractal geometry usually serve quantitatively as a measure for the internal structural disorder and irregularity^15^, which connects back to the basis (Rényi Entropy) on which it is defined^16^. As a result, in addition to geometric objects, the multifractal analysis is also applied to topological and stochastic objects as well and unveils an underlying geometric links to their complexity through an information-theoretic measure^17^, identify geochemical anomalies^18^ and decompose and analyze the particulate matter concentrations^19^. Multifractal analysis is suitable for variables with self-similar distributions on a spatial domain, which can provide insight into the spatial variability of soil parameters^20^, and several multifractal parameters and two spectra have been proposed by pedologists^21^. Multifractal techniques are promising alternatives to single fractal dimensions because they show well-defined scaling properties, and detailed information can be obtained from a distribution^22,23^. Fractal and multifractal soil parameters can provide potential indicators of soil quality influenced by land use and are capable of characterizing spatial and temporal differences in different land-use patterns^24,25^. These parameters have been applied to soil erosion^26^, layered sediments^27^ and changes in the carbon and nitrogen distributions in soil^9,28^; however, only a few studies have applied the fractal dimension (*D*_m_) to soil PSDs during a biological invasion.

Soil organic matter (SOM) and nitrogen are important components of soil quality and are widely used in soil quality evaluations^29^. SOM plays unique roles in the maintenance and recovery of soil functions, physical integrity, fertility and environmental quality^30^. In addition, soil quality can affect the structure of vegetation, the growth of plants and litter decomposition and return, which can lead to further impacts on SOM^31^ and nitrogen contents^32^. Decreases in nitrogen content can reduce soil fertility, nutrient supplies, porosity, and permeability^33^. In contrast, the succession of vegetation causes changes in soil properties, such as in SOM and nitrogen contents^32^. Different nutrients are needed for the normal growth of plant species, and changes in soil properties will lead to changes in the forest community^34^. The original species might be replaced by species more suited to the soil properties^35^. Therefore, when an invasion occurs, it changes the soil environment in the habitat, weakens the coordination between local species and the soil, and results in succession. Many studies have shown that SOM and nitrogen are significantly correlated with the fractal dimension of soil particle size^24,36^, with fine soil particles capable of retaining more organic carbon^37^. Therefore, studying the relationships between soil particle size and soil nutrients is helpful for developing a better understanding of soil characteristics.

*Robinia pseudoacacia* is a pioneer tree species native to North America, and it grows under a wide range of climate conditions^38^. This tree has been successfully cropped for biomass production and planted in post-mining areas characterized by water limitation and harsh edaphic conditions. Because this species reproduces quickly and is able to live in symbiosis with rhizobia and thus fix atmospheric nitrogen, it has potential as a key species for short-rotation plantations on marginal land. *Robinia pseudoacacia* can significantly improve the conservation of water and soil^39^, improve the physical structure of soil^40^, increase the anti-erosion properties of soil^41^, and enhance the nitrogen mineralization rates of soil^42^. *Robinia pseudoacacia* has expanded its range throughout North America and now occurs in all contiguous states and throughout southern Canada. This species is officially listed as invasive in Connecticut and Massachusetts and has been described as invasive in other states^43,44^. *Robinia pseudoacacia* is colonized by arbuscular mycorrhizal fungi (AMF), ectomycorrhizal fungi, and soil-borne pathogens^45^, which improve the plant’s ability to regenerate root shoots and disperse^46–48^. Thus, it is now invasive in many parts of the world. At the beginning of 17^th^ century, Hungary introduced *Robinia pseudoacacia*, which is now found over 20% of the country, with approximately 2/3 originating from the regeneration of root shoots. Subsequently, the development of coppice production and the ecological functions of the ecosystems in which it occurs have declined dramatically. In China, *Robinia pseudoacacia* was introduced to Tai’an in the 1920s and gradually became the main tree species of the Taishan Mountain vegetation below 1000 m altitude, where it has seriously restricted the growth of native tree species and led to environmental deterioration. Studies have shown that root regeneration is the mode of population dispersal in *Robinia pseudoacacia*, and under increased interference, it produces a greater number of suckers; thus, its dispersal ability is directly and positively related to soil nutrients^49^. We expect to observe relationships among soil nutrients, PSDs and *Robinia pseudoacacia* invasion; therefore, in this study, we evaluate the ability of this species to invade different habitats based on soil properties to inform our theory of *Robinia pseudoacacia* invasion.

Ordination methods, which are considered robust quantitative analysis techniques, are used to analyze entities as well as their attributes and correlations with environmental variables^50,51^. However, these methods have rarely been used to study PSDs. Gui^52^ was the first to analyze the variations in the characteristics of PSDs and the relationships between PSDs and environmental factors by ordination. Gui indicated that ordination methods could be useful for PSD research and suggested that the combination of fractal measurements and ordination methods could provide comprehensive information on PSDs. When the habitat of native plant communities is similar to the habitat of invasive species, the possibility of invasion occurs^53^. Thus, in this paper, we use fractal-scaling theory to analyze the soil properties of four plantations, the preferred environment of *Robinia pseudoacacia* and the similarities among the four plantation soil environments using ordination methods, which can improve our evaluative indicators of habitat invasibility. The objectives of this study were to 1) assess the effects of forests on soil physical properties and 2) explore the potential of the fractal dimension of soil PSDs as an integrating index for quantifying habitat similarity, thereby providing a theoretical basis for revealing the diffusion mechanism of *Robinia pseudoacacia*.

## Results

### Soil fractal characteristics of the different plantations

**Figure 1:**
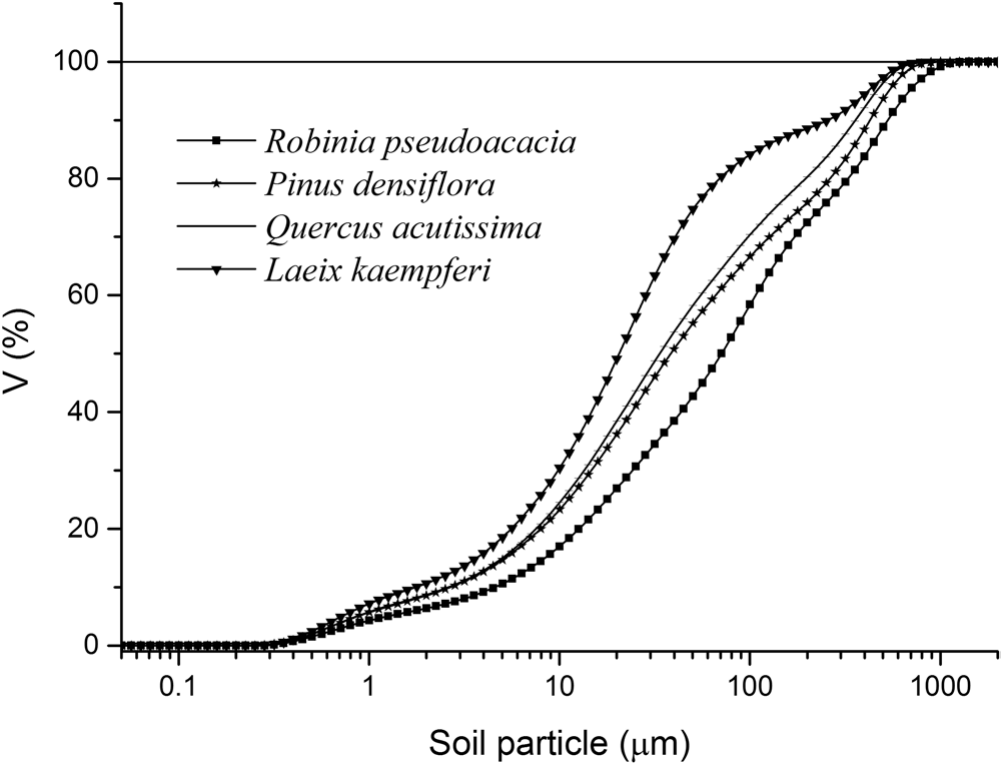
Frequency distribution of volume accumulation content of the soil particles in different plantations.

The soil size particle composition was measured using a laser particle-size analyzer. The cumulative frequency curves of the soil PSDs in the four plantations are shown in Fig. 1. The figure shows that when the cumulative distribution frequency of the soil particle size reached 50%, the particle size of the *Robinia pseudoacacia* (RP) plantation was 0.02–71 µm, the particle size of the *Pinus densiflora* (PD) plantation was 0.02–40 µm, the particle size of the *Quercus acutissima* (QA) plantation was 0.02–35 µm, and the particle size of the *Larix kaempferi* (LK) plantation was 0.02–20 µm. At PSDs of 0.02–200 µm, the cumulative distribution frequencies of the soil particle sizes of the RP, PD, QA, and LK plantations were 72.47%, 75.90%, 80.27%, and 88.54%, respectively.

**Figure 2:**
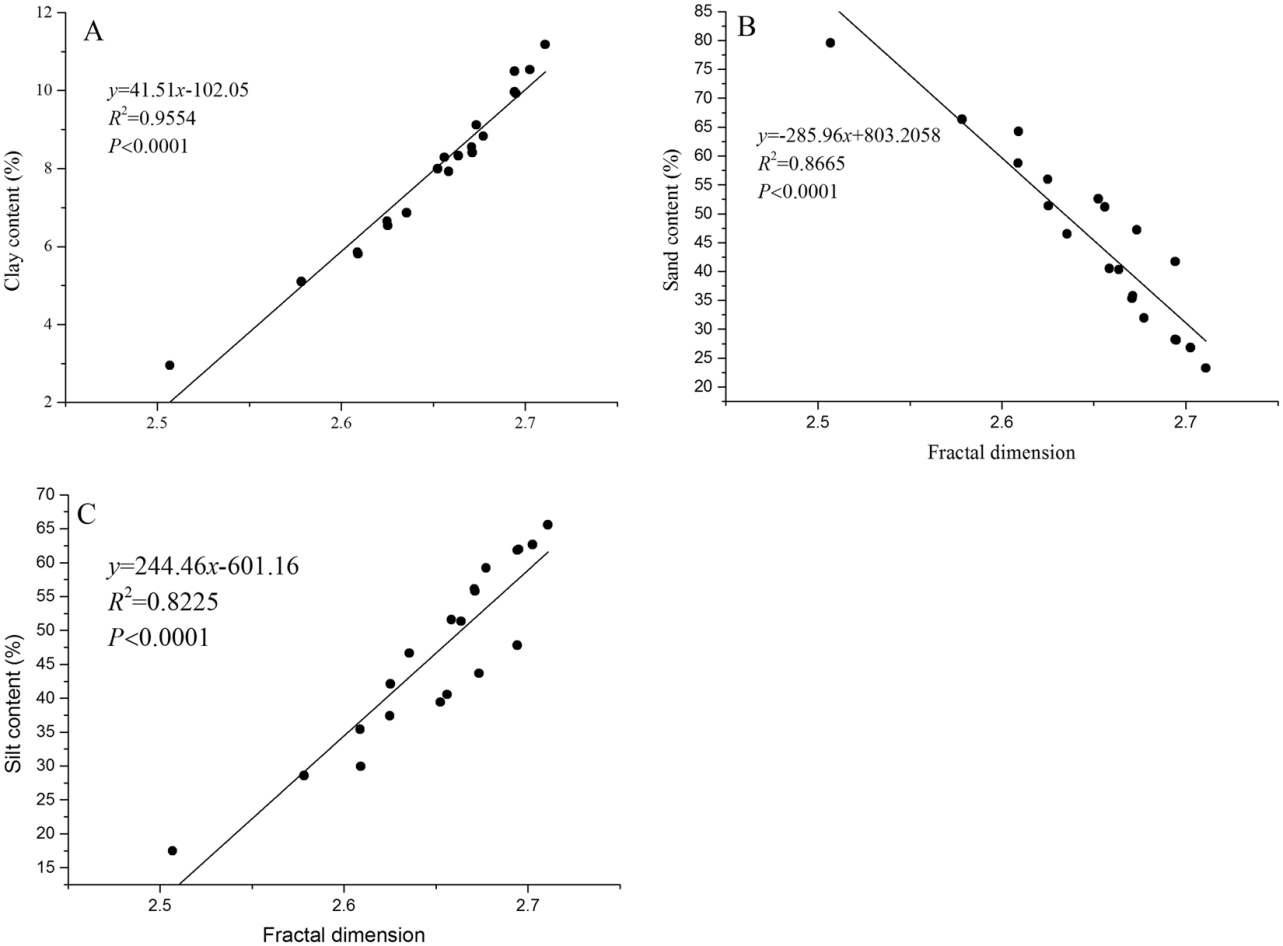
Relationships between soil fractal dimension and (**A**) clay, (**B**) sand, and (**C**) silt content in the 0–20 cm soil layer (n = 20).

The soil PSDs and *D*_m_ values of the sampling sites are shown in Table 1. The PSDs vary considerably among the four plantations. The predominant soil particle size categories are silt and sand, with percentages ranging between 32.01% and 62.24% and 27.67% and 62.52%, respectively. The content of clay is relatively low, at only 5.47–10.09%. For clay, silt and sand, the plantations rank LK > QA > PD > RP, LK > QA > PD > RP, and RP > PD > QA > LK, respectively. These results indicate that for the RP plantation, the content of sand is high, and the contents of clay and silt are low. The QA and PD plantations are similar, whereas the LK plantation presents a different pattern. These results indicate that the RP plants might prefer an environment with a high proportion of sand and thus prefer the soil environments of the PD and QA plantations. Linear regression analyses were performed to determine the strength of the relationships between *D*_m_ and the clay, silt and sand contents in the 0–20 cm soil layer (Fig. 2). The results show that the fractal dimensions of the PSDs were strongly positively correlated with the clay and silt contents (Fig. 2A,C; *R*^2^ = 0.9554 and 0.8255, respectively) and negatively correlated with the sand content (Fig. 2B; *R*^2^ = 0.8665). Soils with higher silt-clay contents and lower sand contents had higher *D*_m_ values.

**Table 1:** Compositions of the soil PSDs and *D*_m_ values in different plantations.

Plantations	Clay (%)	Silt (%)	Sand (%)	*D*_m_	*R* ^2^
*Robinia pseudoacacia*	5.47 ± 1.58c	32.01 ± 10.84c	62.52 ± 12.33a	2.59 ± 0.05b	0.9402
*Pinus densiflora*	8.08 ± 1.89b	44.96 ± 7.52b	46.96 ± 8.81b	2.65 ± 0.04a	0.8952
*Quercus acutissima*	8.22 ± 0.26b	47.81 ± 7.40b	43.98 ± 7.52b	2.66 ± 0.01a	0.8796
*Larix kaempferi*	10.09 ± 1.58a	62.24 ± 2.27a	27.67 ± 3.12c	2.70 ± 0.01a	0.8080

Notes: Values are the means of three replications ± SD. Means within a column followed by different letters are significantly different (*P* < 0.05). An *R*^2^ value below 0.95 is acceptable.

The *D*_m_ values of the soil PSDs for the four plantations were determined using Eq. (1) and the determination coefficients (*R*^2^ values) in Table 2. The *R*^2^ values range between 0.808 (LK) and 0.9402 (RP), and these values are very similar to the experimental data of Liu^8^. These results indicate that for the different plantations, using the power law or single fractal dimension as a descriptor for soils within these PSDs provides sufficient accuracy. The *D*_m_ values range from 2.59 (RP) to 2.70 (LK) and are significant at the 0.05 confidence level, which indicates that the plantations show obvious fluctuations in fractal features. The *D*_m_ values are ordered LK > QA > PD > RP, and significant differences were observed in the *D*_m_ values between the RP plantation and the other plantations. The fractal dimensions of the RP soil were much lower than those of the other three soils. These changes are consistent with those of clay and silt and inconsistent with those of sand, which indicates that the particle size characteristics of the RP plantation are similar to those of the PD plantation.

**Table 2:** Parameters of multifractal spectra of soil particle-size distribution in different plantations.

Plantations	*D*_0_	*D*_1_	*D*_1_/*D*_0_	*Hq(q = 2)*	*Δα*	*Δf(α)*
*Robinia pseudoacacia*	0.921 ± 0.051	0.910 ± 0.019a	0.990 ± 0.044	0.942 ± 0.026	0.953 ± 0.069a	0.943 ± 0.012
*Pinus densiflora*	0.910 ± 0.014	0.903 ± 0.007a	0.988 ± 0.016	0.942 ± 0.004	0.382 ± 0.019c	0.927 ± 0.006
*Quercus acutissima*	0.913 ± 0.017	0.901 ± 0.010a	0.994 ± 0.020	0.945 ± 0.003	0.459 ± 0.020c	0.916 ± 0.037
*Larix kaempferi*	0.887 ± 0.006	0.875 ± 0.004b	0.986 ± 0.009	0.922 ± 0.004	0.760 ± 0.089b	0.934 ± 0.016b

Notes: Values are the means of three replications ± SD. Means within a column followed by different letters are significantly different (*P* < 0.05).

### Soil multifractal characteristics of the different plantations

**Figure 3:**
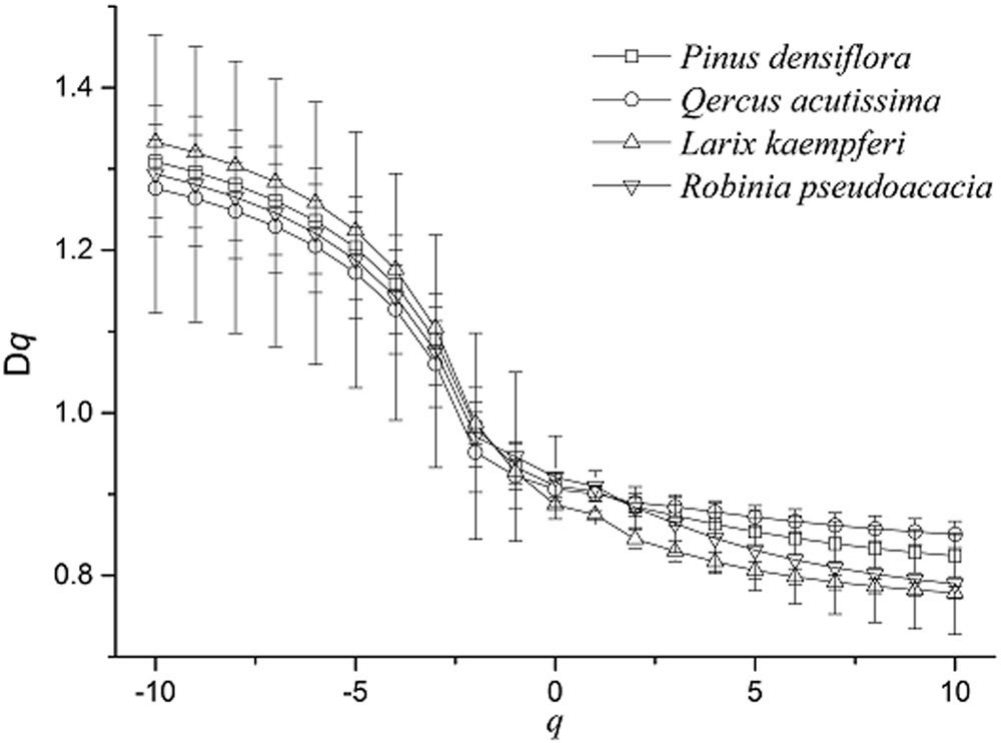
Spectrum curves D_(*q*)_-*q* of generalized dimensions in different plantations.

Based on Eqs (3–6), the Rényi dimension spectra *D*_*(q)*_ of the different plantations were calculated for −10 ≤ *q* ≤ 10 at 0.5 lag increments; they are shown along with their standard error bars in Fig. 3. The calculated *D*_(*q*)_ values indicated that the reported properties are closer to the singular measure spectra than they are to the soft density spectra. This observation suggests that multifractal models can accurately simulate the internal structure of the constructed measures from soil PSDs. This assumption was tested^54,55^ in simulations of the soil PSDs via Iterated Function Systems, and the results indicated the suitability of multifractal measures for modeling these distributions.

As shown in Fig. 3, Rényi spectra of the soil PSDs of *Pinus densiflora*, *Quercus acutissima*, *Robinia pseudoacacia*, and *Larix kaempferi* followed a typical anti-S-decreasing function. When *q* > 0, the decreasing trend of *D*_*(q)*_ slowed, and the dimensions of the different soils approached 0.9. The *D*_*(q)*_ values of *Larix kaempferi* plantation soil changed obviously in the range −10 ≤ *q* ≤ 10, which was more inhomogeneous than that of the other soil types. In addition, the *Quercus acutissima* plantation soil was more evenly distributed.

**Figure 4:**
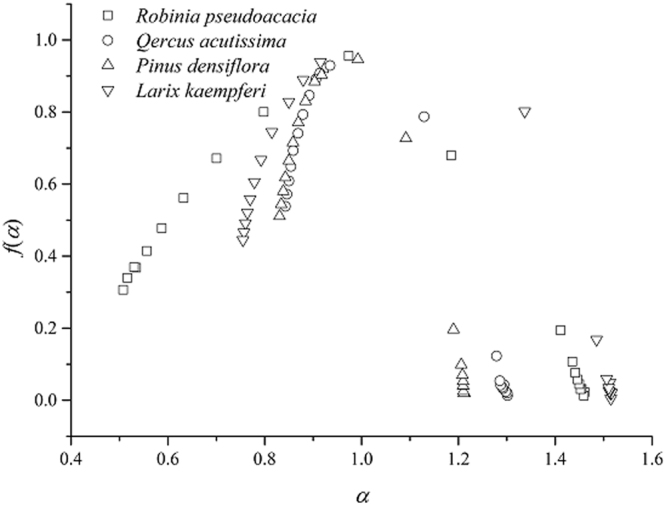
Multifractal spectra (*f*(*α*) versus *α*) for *Robinia pseudoacacia, Pinus densiflora, Quercus acutissima* and *Larix kaempferi*.

The multifractal spectrum provides information about the relative importance of various fractal exponents present in the series. In particular, the width of the spectrum indicates the range of present exponents^56^. Multifractal spectra from different plantations are shown in Fig. 4. Soil particle size distribution *f*(*α*) versus *α* functions were continuous convex functions, indicating that the soil particle distributions from different plantations had heterogeneous properties. This result also indicated that the soil was a complex fractal. The width and shape of a multifractal spectrum can be used to characterize and quantify the properties. A summary of Hurst index values is provided in Table 2, where the range and average of the values characterize the different scenarios contemplated in this work. Here, we focus on the quantification of multifractal spectra amplitudes and symmetries through the difference of the extreme singularities (Δ*α* = *α*_max _− *α*_min_) and the difference of their respective *f*(*α*) values Δ*f*(*α*) = *f*(*α*_max_) − *f*(*α*_min_)^20^. In this way, a higher Δ*α* value indicates higher complexity of the structure studied in the four plantations. All the spectra in Fig. 4 are strongly asymmetric, with the range on the left side of the plot being much smaller than the range on the right side. The Δ*α* value of RP was significantly higher than those of the other plantations, although the Δ*α* values of the PD and QA plantations did not change significantly. These results indicated that the heterogeneity of soil particle composition was higher in the RP plantation. A higher Δ*f*(*α*) value indicates higher asymmetry in the multifractal spectra (right-handed if Δ*f*(*α*) > 0). The results indicate that the four plantations have high asymmetry.

A one-way ANOVA was performed to assess the differences between the treatments for various parameters derived from the plots of the singularity spectrum; *Δα*; Δ*f*(*α*); and the generalized spectrum dimensions, *D*_0_, *D*_1_ and *D*_1_/*D*_0_. The capacity dimension (*D*_0_) was calculated using the box-counting technique. Table 2 shows that the highest *D*_0_ value was observed for the RP plantation (0.921) soil PSDs, whereas lower *D*_0_ values were observed for the LK plantation soil PSDs (0.887). *D*_0_ provides general information about the PSDs system because it represents the dimension of the set of sizes with a non-zero relative volume. *D*_0_ = 1 means that all subintervals are occupied at all scales, whereas *D*_0_ = 0 means that all subintervals are empty^15^. Therefore, the soil PSDs from the RP plantation occurred over a relatively wide range, whereas the PSDs from the LK plantation presented a relatively low range, and no differences among *D*_0_ values were observed.

Considering that the *D*_0_ values reflect the range of a continuous distribution and that the *D*_1_ values express the range of PSDs and measure the homogeneity among fractions at different partition levels, *D*_1_/*D*_0_ was used to describe the heterogeneity in a distribution as suggested by Posadas^14^. As shown in Table 3, the *D*_1_/*D*_0_ values were ordered QA > RP > PD > LK. *D*_1_/*D*_0_ = 1 means that all fractions have equal values at different scales, thus indicating the most heterogeneous distribution. Low *D*_1_/*D*_0_ values reflect a distribution in which irregularities are concentrated.

**Table 3:** Relationship between the generalized Hurst exponent (*Hq*) and the generalized fractal dimension (*Dq*).

Plantation	Regression equation	*R* ^2^	*P* value
*Robinia pseudoacacia*	*Dq* = 0.9736*Hq* − 0.0084	0.974	0.03557
*Pinus densiflora*	*Dq* = 0.9442*Hq* + 0.0336	0.9649	0.03119
*Quercus acutissima*	*Dq* = 0.9768Hq + 0.0154	0.9768	0.02954
*Larix kaempferi*	*Dq* = 0.9753*Hq* − 0.0041	0.9803	0.02761
Total	*Dq* = 0.9643*Hq* − 0.0084	0.9739	0.03071

The *D*_1_/*D*_0_ values of the four plantations ranged from 0.986 to 0.994, which indicated a heterogeneous distribution.

The values of the entropy dimension (*D*_1_) were ranked RP > PD > QA > LK. *D*_1_ provides a measure of the heterogeneity of PSDs^17^. When the *D*_1_ value is higher, the soil’s PSDs are more heterogeneous, the PSD range is wider, and the measures are more homogeneous among regions over all sets. Significant differences in *D*_1_ values were detected among the four plantations at the 95% confidence level. The *D*_1_ values of the soil PSDs from the LK plantation were significantly lower than those of the other three plantations. The *Δα* values of the RP plantation were significantly higher than those of the other plantations, and there were no significant relationships among the four plantations.

At different time scales, the Hurst exponent was greater than 0.5, which indicated that the time series of the four plantations are non-random. persistent, and take on long-range dependence; in general, the time series of particle size distribution showed an upward or downward trend. Thus, in the future, it will improve. In all cases, we found a linear relationship between *Hq* and *Dq*. The *R*^2^ values were consistently higher than 0.96 at *P* < 0.05 (see Table 3). The *P* value of the relationship for the LK plantation was lowest, and the *R*^2^ value was highest for this plantation. Overall, the generalized Hurst exponent was linearly related to the generalized fractal dimension (*R*^2^ = 0.9739, *P* = 0.03071).

### Relationships between multifractal parameters and soil properties

SOM, available nitrogen (AN) and available phosphorus (AP) were selected as the soil quality indicators. Table 4 shows that the contents of SOM, AN and AP in the RP plantation were higher than those in the other three plantations (21.40 g/kg, 115.92 mg/kg and 37.76 mg/kg, respectively) and the differences were significant at the 0.05 level. These results indicate that planting RP can improve soil quality and show that the dispersal of RP is strongly correlated with SOM and AN^42^. The SOM and AN values were ordered as follows: RP > PD > LK > QA.

**Table 4:** SOM, AN and AP contents in the 0–20 cm layer at each plantation.

Plantation	AN (mg/kg)	AP (mg/kg)	SOM (g/kg)
*Robinia pseudoacacia*	115.92 ± 24.86a	37.76 ± 9.75a	21.4 ± 4.37a
*Pinus densiflora*	86.13 ± 9.69b	18.13 ± 4b	13.56 ± 2.28b
*Quercus acutissima*	63.73 ± 13.37b	13.37 ± 6.8b	9.32 ± 2.02c
*Larix kaempferi*	83.89 ± 4.31c	11.29 ± 2.08b	11.42 ± 2.39bc

Notes: Values are the means of three replications ± SD. Means within a column followed by different letters are significantly different (*P* < 0.05).

A simple correlation analysis was performed to establish the relationships between *D*_m_ and the sand, silt, and sand contents. The results indicate that the fractal dimension of the PSDs was highly significantly and positively correlated with the contents of clay and silt (*R*^2^ = 0.9554 and 0.8255, respectively) and negatively correlated with the content of sand (*R*^2^ = 0.8665), which indicates that the removal of fine particles (clay and silt particles) results in decreased *D*_m_ values. The decrease in *D*_m_ indicates a decrease in fine particles and their accumulation in coarser fractions.

To analyze the relationships between the different parameters, a simple correlation analysis was conducted and the correlation between multifractal parameters and soil properties are shown in Table 5. The multifractal parameters were consistent in reflecting the multifractal law of soil particle composition. Among the soil properties, SOM was positively and significantly correlated with AN, AP, Δ*f*(*α*) and Δ*α*, which are indices of soil quality that reflect the soil nutrient conditions. Here, SOM was significantly positively correlated with *D*_0_, whereas it showed a weak or no correlation with *D*_1_/*D*_0_ and *D*_1_. The highest correlation coefficient was observed for SOM and *D*_0_. In addition, SOM increased as *D*_m_ decreased.

**Table 5:** Relationships between soil chemical properties and soil physical properties.

	Clay (%)	Silt (%)	Sand (%)	*D*_m_	*D*_1_/*D*_0_	*D*_0_	*D*_1_	*Δf(α)*	*Δα*
AN	−0.447*	−0.407	0.418	−0.418	0.202	0.432	0.407	0.258	0.575**
AP	−0.734**	−0.611**	0.636**	−0.731**	0.073	0.507*	0.375	0.29	0.547*
SOM	−0.557*L	−0.535*	0.545*	−0.545*	0.013	0.568*	0.386	0.452*	0.551*

^*^Pearson correlation is significant at *P* < 0.05; **Pearson correlation is significant at *P* < 0.01.

### Habitat similarity analysis

**Figure 5:**
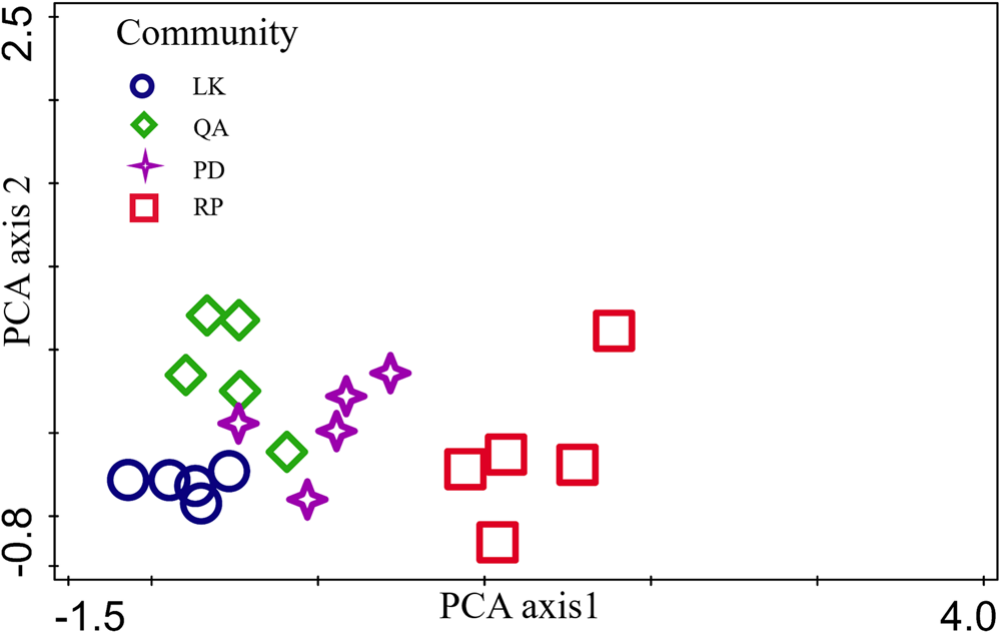
Two-dimensional PCA ordination diagram of plantation soil properties.

A direct gradient analysis was used to describe the soil habitat similarity based on changes in the habitat. The four plantations were classified using Canoco 5.0, and detailed information was obtained on the clay, silt, and sand contents; *D*_m_; AN; AP; SOM; *D*_1_/*D*_0_; *D*_0_; *D*_1_; Δ*f*(*α*) and Δ*α*. The plantation distribution patterns were analyzed by PCA. The eigenvalues of the four PCA axes were 0.7130, 0.1450, 0.0823 and 0.0285. Figure 5 shows the PCA ordination diagram based on the first and second axes. In Fig. 5, the values for each plantation occur within a limited range and are clearly separated from those of the other plantations. The RP plantation was similar to the PD plantation and dissimilar to the LK plantation, and cross-correlations were observed among the PD, QA and LK plantations. These results indicate that the RP and PD trees share similar habitats.

## Discussion

Soil PSDs are commonly used to classify soils and estimate various related soil properties^57^. Fractal geometry has been increasingly applied as an effective tool for describing the structure, dynamics, and physical processes of soil, thereby facilitating a better understanding of the performance of a soil system^1,8^. Therefore, in this paper, we studied the fractal characteristics of soil in different plantations. Our analyses revealed significant positive correlations between *D*_m_ and clay and silt contents (*R*^2^ = 0.9554 and *R*^2^ = 0.8255, respectively) and a significant negative correlation between *D*_m_ and sand content (*R*^2^ = 0.8665). These results indicate that the soil clay content affects the *D*_m_ value, which is also affected by the maximum size of the soil particles. These findings are similar to those of other studies^8,9,27,58,59^. The results indicate that smaller particle sizes reflect a greater spatial filling capacity of the soil, which corresponds to higher fractal dimension values based on pore geometry^13^. In addition, we found that the sand content of the RP plantation was significantly higher than the sand contents of the QA, PD and LK plantations (*P* < 0.05), whereas the clay and silt contents showed the opposite trend (*P* < 0.05). These results indicate that the RP plantation achieved a lower *D*_m_ value due to a higher sand content. Thus, RP might prefer soil environments with higher sand contents and might invade habitats with such contents more easily than can other plants.

The fractal dimension is a sensitive and useful index for quantifying changes in the properties of soil^60^, and several linear relationships have been observed between fractal dimensions and soil properties^61^. The proportion of each fraction has a dominant influence on many soil properties. The clay fraction constitutes the reactive fraction of the soil, whereas the sand and silt fractions are relatively inert^62^ and can partially reflect the stability of a plant community^59^. Therefore, the relationships between *D*_m_ and soil physical-chemical properties were studied here. The results show that soil PSDs can influence the soil water infiltration and moisture retention and the availabilities of water and nutrients to plants^63^, and many model simulations of SOM decomposition and formation have shown that soil PSDs can also control the SOM dynamics^64^. When the fractal dimension is higher, the clay content is higher, and a larger surface area, a stronger bond, and more nutrients are observed^65^; these factors significantly improve the SOM^9^. In addition, higher SOM is beneficial for the retention of fine soil particles^26,37,66^, whereas *D*_m_ is negatively correlated with SOM^59^. Our results indicated significant and negative correlations between *D*_m_ and each of the clay and silt contents, SOM and AP, whereas significant and positive correlations were observed among sand content, SOM and AP. The fractal dimension of soil PSDs can quantify the properties of soil particle composition, which reflects the size of soil particles and the relationship between particle size and the degree of soil thickness. The fractal dimension of soil texture can be characterized from sand to fine changes. This finding may reflect correlations between the PSDs and the microbial community composition^67,68^ and may demonstrate the responses of plant and forest communities to soil heterogeneity^69^. The latter is a basic element for competitive and facilitative interactions between plants^70^ and can determine the patterns of plant and community distributions^71^. The root structures of various plant species might be a source of differences in the fractal dimension^72^. The RP plantation at Mountain Tai presents a stronger diffusivity and a developed root system that shows increases in residual inputs and the concentrations of fine roots with abandonment age^73,74^. Additionally, these plantations release large amounts of soil nutrients^75^, which improves the soil nutrient conditions. Although the LK plantation had the highest *D*_m_ value, this value was correlated with the content of allelochemicals in the root exudates, and lower nutrient allelochemicals contribute greatly to the cohesion of fine soil particles^60^. *D*_m_ reflects the spatial filling capacity of soil based on the distribution of soil particles^33,76^, although reductions of SOM and TN levels will result in decreased soil fertility, soil nutrient supplies, soil porosity, soil penetrability and soil productivity^77^. Then, *D*_m_ is improved^3^.

The heterogeneity of multifractal spectra can be assessed in different ways^78^. For example, the curvature and symmetry of the *f*(*α*) spectra provide information on the heterogeneity, which can also be assessed by the magnitude of changes around *D*_0_ in both *f*(*α*) and *α*^14^. The width of the *f*(*α*) spectrum is defined as the difference between the most positive and negative moments used in the evaluation of the singular spectra and the moment order zero (Δ*α*). This width may be considered as an indicator of symmetry/asymmetry of a multifractal system^79^. For the independent random process, with no correlation among samples, *H*_*q*_ = 0.5. The observational time series is persistent for *H*_*q*_ > 0.5, whereas the sequence shows anti-persistent behavior for *H*_*q*_ < 0.5^80^. The *H*_*q*_ values of the four plantations were higher than 0.5, indicating they are persistent. The generalized dimension, *D*_*q*_, is a constant in the case of scale invariant distributions but changes with *q* for multifractal measures. A *D*_1_ value close to *D*_0_ should be an indication of a measure distributed over all the study scales, whereas a *D*_1_ value close to 0 should reflect irregularities, indicating that most of the measure is concentrated within a small size domain of the study scale. The ratio *D*_1_/*D*_0_ may be considered a measure of evenness in the context of multifractal spectra^23^. Thus, low values of this parameter reflect a distribution in which irregularities are concentrated, whereas high values indicate the opposite. *D*_0_, *D*_1_, and *D*_1_/*D*_0_ may be seen as indicators of abundance, dispersion and evenness, respectively^62^. The *D*_0_ values of the RP, PD, QA, and LK plantations were 0.921, 0.910, 0.913, and 0.887; these values indicate four degrees of abundance, which in this case alludes to the scaling of the number of cells containing some particle volume and further suggest similitude of the studied PSDs. The capacity dimension, *D*_0_, ranged from 0.887 to 0.921, the entropy dimension, *D*_1_, ranged from 0.875 to 0.910, and the ratio *D*_1_/*D*_0_ ranged from 0.986 to 0.994. These values indicate somewhat diverse scaling properties among the soil samples, and the ranges of values suggest that none of these single parameters should be used to discriminate among measures, specifically PSDs in our case study.

The multifractal parameters were significantly correlated with SOM^81^. Therefore, in this study, a multifractal analysis of the samples was conducted. As shown above, the *D*_0_, *D*_1_, and *D*_1_/*D*_0_ values reflected different information on the soil PSDs, and plantation type had an influence on the *D*_0_, *D*_1_, and *D*_1_/*D*_0_ values. The fractal dimensions (*D*_1_, *D*_m_) that mainly reflect fine soil particles are highly correlated with the SOM content of soils. The fractal dimension *D*_0_ can provide useful information on the coarse particle fraction^82^. Table 6 shows that significant correlations were not present between SOM and *D*_1_ or *D*_1_/*D*_0_, although a significant correlation was observed between *D*_0_ and SOM. These findings are inconsistent with those of Wang^81^ and Sun^83^, who found that *D*_0_ was correlated with the coarse particle fraction^82^. Furthermore, in the present study, the coarse content was positively correlated with the SOM (Table 5).

**Table 6:** Regeneration of tree species in the *Pinus densiflora* planation.

Species	Cover/%	Frequency/%	Density/plants/hm^2^
*Diospyros lotus*	25	30	5000
*Robinia pseudoacacia*	10	17	2660
*Broussonetia kurzii*	20	3.3	670
*Pistacia chinensis*	5	7	670
*Quercus acutissima*	5	3.3	670

Invasibility is an emergent property of invaded ecosystems and their established species, and it affects only the extinction rates of the invaders and not their immigration rates^84,85^. Recently, studies have focused on the relationships between communities and invasibility^86,87^, but the findings are inconsistent^87^. Studies have shown that local environmental conditions can be used to predict plant invasions^88,89^, and niche-based modeling can be used to predict the risk of alien plant invasions at a large scale^85,90–92^. When additional resources are available, a community is more vulnerable to invasion^93^; thus, soil nutrients can affect the invasibility of a plant community. This phenomenon likely offers alien plants a dominant advantage in competition with native plants, and it promotes the likelihood of invasion by alien plants^94,95^. Therefore, we evaluated the soil properties of four plantations (Tables 1–3). In previous work, the degree of similarity between a new environment and a native environment was assessed and found to be beneficial for understanding community invasibility^96^. RP plantations are mainly rooted in Mountain Tai and show strong correlations with the characteristics of the soil; therefore, we can evaluate the community invasibility based on the soil properties. The ordination method can be applied to analyze the variation characteristics of PSDs and the relationships between PSDs and environmental factors^45^. Community invasibility can be used to evaluate the degree of invasion of a region or community. We found that the characteristics of the RP plantations were strongly correlated with those of the PD plantations (Fig. 5), which indicates that RP plants are more likely than are other plants to invade PD habitat. We also found that RP had regenerated in the PD plantation (Table 6), which is consistent with the abiotic suitability hypothesis^97^.

## Conclusions

We studied the fractal dimension of soil PSDs in soils within plantations of different tree species (PD, QA, RP and LK) and evaluated the possible factors that indicate invasion. The fractal and multifractal characteristics of 20 soil PSDs were studied. The results showed that a law relationship could be applied to all the soil PSDs. *D*_m_ varied from 2.59 to 2.70 among the different plantations and reached the highest values in the LK plantations. *D*_m_ was negatively correlated with sand content and positively correlated with the silt and clay contents. A significant and negative correlation was observed between *D*_m_ and both SOM (*P* < 0.05) and AP (*P* < 0.01). These results suggest that *D*_m_ can be used to characterize the uniformity of soil texture to a certain extent as well as the soil fertility characteristics. The *D*_1_ and *D*_1_/*D*_0_ values were not significantly correlated with SOM, whereas the *D*_0_, Δ*α*, and Δ*f*(*α*) were each significantly correlated with SOM.

Our analyses of the PSDs and soil physical-chemical properties of the different plantations in the study area indicated that the PD, QA and LK plantations presented similar habitats. Compared with the other plantations (PD, QA and LK), the RP plantation had more nutrients, higher *D*_0_ and *D*_1_ values, and lower *D*_m_ values. The PCA ordination showed that the habitat of the PD plantation is similar to that of the RP plantation, which indicates that in the same environment, RP is more likely than other species to invade a PD habitat.

## Materials and Methods

### Study area

The study was conducted at the Yaoxiang Forest Farm, in Mount Tai, China. The farm is located on the border of the southern suburbs of the cities Ji′nan and Tai′an and north of the main peak of Mount Tai. The geographical coordinates are 117°10′E and 36°17′N, and the study area is 12 kilometers long and 5 kilometers wide from north to south, with a total area of 1210.2 hectares. The region has a warm temperate continental monsoon climate. The annual average temperature is 10.8 °C; the maximum temperature is 34 °C, and the minimum temperature is −24 °C. The average annual precipitation is 900–1000 mm, and the altitude above sea level ranges from 400–956 m. The soil types are typical of mountain brown terrain, and the soil thickness is 15–90 cm. After years of reforestation, the forest farm consists almost entirely of plantations, which were mostly planted in the 1950s, and includes a small amount of shrub forest.

### Soil sampling and analysis

The experimental plot was established in a standard sample of *Pinus densiflora* (PD), *Quercus acutissima* (QA), *Robinia pseudoacacia* (RP) and *Larix kaempferi* (LK) plantations at the Taishan Forest Ecosystem Research Station. The sample plots are described in Table 7. The soil samples were collected from the 0–20 cm soil layer in all plots using the five-spot-sampling method, with five replicates of each sample, for a total of 20 samples. After the removal of residual litter, the samples were air dried, passed through a 2-mm screen, and then returned to the lab for analysis. Each sample was divided into two parts, with one part used to analyze the soil particle composition and the other part ground and passed through a 0.25-mm screen and used to determine the contents of SOM and available nutrients.

**Table 7:** Basic information on the soil sampling sites.

Plantations	Slope(°)	Aspect	Altitude /m	Tree height/m	DBH/cm	Density/ha	pH	Soil thickness/cm
*Robinia pseudoacacia*	40	North	720	15.84	23.92	356	4.94	24.50
*Pinus densiflora*	26	North	703	12.48	22.62	470	4.67	35.33
*Quercus acutissima*	23	South	730	13.76	20.41	551	5.00	26.08
*Larix kaempferi*	29	North	718	15.30	18.25	640	4.98	32.00

The soil PSDs were measured via laser diffraction with a Mastersizer 2000 Particle Size Analyzer (Malvern Instruments, Malvern, England), which has a range of 0.02–2000 μm and repeated measurement errors of less than 2%. From each sample, 0.5 g samples were soaked for 36 h in hyperpure water, stirred, and then heated, and the supernatant was subsequently removed. Each sample was dispersed for 30 s using an ultrasonic wave, and the percentage of the soil particle-volume fraction was determined. The results were presented using the US standard for classifying soil particle size: 0–0.002 mm, 0.002–0.05 mm, 0.05–0.1 mm, 0.1–0.25 mm, 0.25–0.5 mm, 0.5–1 mm, and 1–2 mm. Each sample was then described in terms of the percentages of clay (<0.002 mm), silt (0.002–0.05 mm) and sand (0.05–2 mm). The SOM was determined using the potassium dichromate oxidation method, the AN was determined using the Conway method, and the AP was determined using the double acid extraction method. Each sample was analyzed in duplicate, and the mean values were calculated. This work was conducted based on the forestry standards described in the “Observation methodology for long-term forest ecosystem research” of the People’s Republic of China.

### Soil fractal model theory

Based on the soil texture classification system of the US and the volume fractal dimension formula deduced by Gao^7^, the fractal scaling of PSDs was performed as follows. The cumulative number of soil grains (r) greater than a characteristic size (specific measuring scale, R) is set to be N (r > R), and the cumulative volume distribution of soil grains (r) smaller than the specific measuring scale, R, is set to be V (r < R). Then, the values of N and V will be proportional to R^D^ and R^3-D^, respectively. The exponent D can be easily determined based on the relationship between R and N or V. The fractal fragmentation can be quantified based on the relationship between the number and size in a statistically self-similar system:

1 $${\rm{N}}({\rm{X}}\ge {x}_{i})={\rm{k}}{x}_{i}^{-D}$$

where N(X > x_i_) is the cumulative number of objects or fragments X greater than the i-th characteristic size x_i_, and k is the number of elements at a unit length scale. However, the relationship given by Eq. (1) is not convenient, and errors can be introduced in the calculation. The applicability of Eq. (1) to PSD analysis is also limited because N values are unavailable in conventional PSD data. Thus, an estimation of D for soil PSD is used here, and the derived equation is as follows:

2 $$\frac{{V}_{(r < R)}}{{V}_{T}}={(\frac{R}{{\lambda }_{V}})}^{3-D}$$

where *V* is the cumulative volume of particles of size *r* and less than *R*; *V*_*T*_ is the total volume; *R* is the mean particle diameter (mm) of the *R* size class; *λv* is the mean diameter of the largest particle; *D* is the fractal dimension; and *V*(*r* < *R*) is the sum of objects with fragments less than a characteristic size. The mean particle diameter is the arithmetic mean of the upper and lower sieve sizes. Based on the logarithm of both sides of Eq. (2) and the linear regression between log (V_(r<R)_/ V_T_) and log(R/λ_V_), the value of 3-D can be determined^20^.

### Multifractal analysis

Multifractal sets can be characterized based on the Rényi dimensions of the *q*th moment orders of distribution, *D*_(*q*)_, which were defined by Rényi *et al*.^98^ and Hentschel *et al*.^99^. Based on the measurement interval of the laser particle size analyzer (*I* = [0.02 µm, 2000 µm]), the 100 subintervals are *I*_*i*_ = [*φ*_*i*_, *φ*_*i*_ + 1], *i* = 1, 2, …, 100; $\sum _{i=1}^{100}vi=100$, *v*_1_, *v*_2_, …, *v*_100_; where *v*_i_ is the soil particle size volume percentage of *I*_*i*_, and *φ*_*i*_ is the measured soil particle size from the laser particle size analyzer. According to the standard particle size division methods for the laser particle size analyzer, log(*φ*_*i*_ + 1/*φ*_*i*_) is constant across the measurement interval of *I* = [0.02 µm, 2,000 µm]. To meet the requirements of the multifractal method, ψ_i_ = log(*φ*_*i*_/*φ*_*1*_) (with *i* = 1, 2, …, 100) was changed. Next, we obtained a new dimensionless interval of *J* = [0, 5], which has 100 equidistant subintervals, Ji = [*ψ*_*i*_, *ψ*_*i*_ + 1], i = 1, 2, …, 100. In the interval *J*, 2^*k*^ same size subintervals were used (ε), with ε = 5 × 2^*−k*^. Each subinterval contained at least one measured value within a k range of 1 to 6^29^.

Multifractal measures can also be characterized by scaling the *q*th moments of [*P*_*i*_] distributions^94^ expressed in the following form:

3 $${\mu }_{i}(q,\delta )=\frac{{\mu }_{i}{(\delta )}^{q}}{{\sum }_{i=1}^{n(\delta )}{\mu }_{i}{(\delta )}^{q}}$$

The Rényi dimension *D*_*q*_ is a monotonous decreasing function for all real *q* values within the interval [−∞, +∞]. Parameter *q* acts as a scanning tool that scrutinizes the denser and rarer regions of the measure µ^23,100,101^. For *q* ≫ 1, regions with a high degree of concentration are amplified, whereas for *q* ≪ −1, regions with a small degree of concentration are amplified.

The generalized fractal dimensions or Rényi dimensions can be calculated as follows:

4 $${\rm{D}}(q)=\mathop{\mathrm{lim}}\limits_{\delta \to 0}\frac{1}{q-1}\times \frac{{\rm{lg}}[{\sum }_{i=1}^{n(\delta )}{\mu }_{i}{(\delta )}^{q}]}{{\rm{lg}}\,\delta }(q\ne 1)$$

5 $${D}_{1}=\mathop{\mathrm{lim}}\limits_{\delta \to 0}\frac{{\sum }_{i=1}^{n(\delta )}{\mu }_{i}(\delta ){\rm{lg}}\,{\mu }_{i}(\delta )}{\mathrm{lg}\,{\rm{\delta }}}(q=1)$$

The singularity strength α(*q*) and singularity spectrum *f*(*α*) are as follows:

6 $${\rm{\alpha }}(q)=\mathop{\mathrm{lim}}\limits_{\varepsilon \to 0}\frac{{\sum }_{i=1}^{N(\varepsilon )}{\mu }_{i}(q,\varepsilon ){\rm{lg}}\,{\mu }_{i}(\varepsilon )}{{\rm{lg}}\,\varepsilon }$$

7 $$f(\alpha (q))=\mathop{\mathrm{lim}}\limits_{\varepsilon \to 0}\frac{{\sum }_{i=1}^{N(\varepsilon )}{\mu }_{i}(q,\varepsilon ){\rm{lg}}\,{\mu }_{i}(q,\varepsilon )}{{\rm{lg}}\,\varepsilon }$$

The graph of *f*(α) versus α is referred to as the multifractal spectrum and typically has a parabolic concave downward shape, with the range of α values increasing with increasing heterogeneity of the measure. A homogenous fractal exhibits a narrow *f*(α) spectrum. The *f*(α) spectrum and the generalized dimensions contain the same information, both characterizing an interwoven ensemble of fractals of dimension *f*(α_i_)^20^.

where *µ*_i_(δ) is the volume percentage of every subinterval, δ is the same size subinterval, *q* is the given parameter, and *D*_(*q*)_ is the information entropy. When *q* = 0, *D*(*q*) = *D*_0_ (*D*_0_ can measure the span of the soil PSDs). When *q* = 1, *D*_(*q*)_ = *D*_1_ (*D*_1_ provides the irregular degree of soil PSDs). *D*_1_/*D*_0_ can measure the degree of heterogeneity of the soil PSDs^51^. *D*_2_ is mathematically associated with the correlation function and related to the Simpson diversity index. The relationship among *D*_0_, *D*_1_, and *D*_2_ can be defined as follows:

$${D}_{2}\,\leqq \,{D}_{1}\,\leqq \,{D}_{0}$$

where the equality *D*_2_ = *D*_1_ = *D*_0_ occurs only if the fractal is statistically or exactly self-similar and homogeneous^52^.

### Multifractal Detrended Fluctuation Analysis (MDFA)

MDFA is thoroughly described in Kantelhardt^102^. In the basic approach, time series are first sub-divided into smaller segments from which are subtracted a least-squares best-fit polynomial of a chosen order to remove the artifacts created by non-stationarities in the time series. A method similar to the moment is then applied to the resulting detrended series. MDFA is described in detail in Salat^103^ and Chamoli^104^. *H*_*q*_ is the generalized Hurst exponent and characterizes the long-/short-range dependence structure in the series^102^. The variation in *H*_*q*_ with *q* is useful for understanding the different scaling of small and large fluctuations. For multifractal time series, the scaling behavior of the large fluctuations is characterized by the values of *H*_*q*_ for positive *q* values. The scaling of the small fluctuations is characterized by the values of *H*_*q*_ for negative *q* values^104^.

### Principal Component Analysis

Principal component analysis (PCA) is a general unconstrained linear method widely used for vegetation pattern analysis. The soil parameters of each plantation were used for the PCA. The soil parameters included the multifractal parameters, PSDs, *D*_m_ and soil properties. The ordination diagram of species and environmental variables derived from the PCA optimally displays the variation of the object composition in connection with the environmental factors. In our analysis, PCA was performed with the four plantations, and the particle-size fractions and AN, AP, SOM contents were included as environmental variables. The plantations were classified using Canoco 5.0 software, and their distribution patterns in the study area were analyzed by PCA.

### Data analysis

A one-way ANOVA was performed to evaluate the effects of plantation on the soil fractal and multifractal parameters, soil AN, soil AP and SOM. The Duncan procedure was used to separate the means of these variables at the *P* < 0.05 level. A correlation analysis was performed to determine the relationships between the multifractal parameters and the quantitative environmental variables using SAS. In this paper, we introduce the method of community ordination to evaluate the similarity of the four plantations based on the soil properties, and the distribution patterns of soil properties in the study area were analyzed via a PCA, which is a general unconstrained ordination method for vegetation pattern analysis, using Canoco 5.0.
